# Some Changes in Dental Practice Made Necessary by Modern Science

**Published:** 1905-08-15

**Authors:** C. M. Wright


### SOME CHANGES IN DENTAL PRACTICE MADE NECESSARY BY MODERN SCIENCE.

C. M. WRIGHT, D.D.S.

Read before the Eastern Indiana Dental Society, May 3, 1905.

If we could draw a picture of the daily practice of the dentist of fifty years ago, and compare it with the practice of the up-to-date dentist of to-day, we should find that the difference between the two pictures would not be so marked as to cause great astonishment on the part of the beholder. The modern appliances and conveniences for saving labor and accomplishing results might attract attention. The machines would call forth praise. The contrast would be between the instruments of that time and this, rather than between what we understand as the "practice." There has been no change in the numbers or character of the diseases of the oral cavity. No malady has *ansen de novo* during the past fifty years. At that time caries and erosions of the teeth, acute and chronic inflammations of the gums and chronic suppurations from the tooth sockets were as prevalent as they are to-day.

If we take down from our library shelves the dust covered volums of Delabare, Jourdain, Bond, Harris and others of the translations from the French, and the text books of that day, we shall find accurate descriptions of tumors, cysts and inflammations of the jaws and of caries, erosions, malformations mal-positions of the teeth. We shall also find interesting dissertations on the diseases of the dentinal pulp, of alveolar abscess and of exostoses of the roots; of deposits of different kinds of tartar and of loosening of the teeth in their sockets. Epulides, necroses and fractures of the maxillae are clearly described.

The daily practice of the average dentist consisted of a great variety of important operations on the teeth themselves and for replacing them by artistic artificial substitutes when they were absent. The tartars were removed by specially designed scalers, some of these being instruments of exquisite design, with pearl handles in which jewels were set. After the operation of scaling, the teeth were polished with orange wood sticks—the short pieces fixed in "portes"—and loaded with water and ground pumice, precisely *a la mode du* Dr. D. D. Smith of to-day. Loose teeth and roots, and roots with chronic abcesses were deftly extracted; remaining teeth were examined with fine explorers and carious spots in the crowns of firm teeth were excavated and filled with gold or tin foils, amalgam (this always under protest), gutta-percha or oxy chlorid of zinc—called at that time "os-artificial."

Incline planes made of silver or gold plate, the screw, the wedge and elastic bands were employed for regulating the front teeth after free spaces had been made by the extraction of bicuspids or sixth year molars. Exposed pulps were devitalized with arsenic or "cobalt" and removed with broaches and the root canals filled with cotton and creosote, tin or gold foils, silk thread, wood slivers, oxy-chlorid, etc.

Superficial caries and erosions were treated with nitrate of silver; sensitive dentin with hot creosote, oil of cloves, nitrate of silver, etc. Hand instruments were employed exclusively and in the forms of chisels, excavators and drills were used for cavity preparation. Files in great variety of shapes and thicknesses were freely employed for reparating and dressing the teeth and in finishing the metal fillings. Forceps and elevators were important instruments in every dentist's case, for the demand was greater for the operation of extraction at that time than at present in most communities. Teeth were extracted when they ached, when they were broken, when they were loose and when they stood in the way of a good arrangement of artificial teeth. The constructing and adapting of artificial substitutes for missing teeth was a major operation in those days.

Incisors were frequently replaced by natural or porcelain crowns mounted on hickory pegs forced into the roots after their preparation by specially designed half-round files and drills. These operations were as perfect esthetically, *i. e.*, in their concealment of art and restoration of natural appearance as anything that is done to-day in this line. The faulty treatment of the canals and the neglect of thorough emptying of the putrescent contents, the lack of disinfections and sterilizations as known and practiced to-day cast this operation into disrepute and for years the prejudice against pivot teeth clung to the people. This prejudice affected for a while the dentist, who willingly substituted forceps and plates in thousands of cases which to-day would be considered most favorable for crowning. At that time, also, transplantation of a tooth from one patients mouth to anothers, and the replanting of teeth that had been knocked out or extracted by mistake was not uncommon.

What do we do to-day that the founders of American Dentistry and these early practitioners did not do? Bridges!

In Geo. Washington's time teeth carved from ivory or cut down from sheep or calves teeth were suspended from one sound tooth to another by ligatures of wire, making a suspension wire bridge instead of the more solid pier bridge of to-day. Skill and ingenuity of the highest order was displayed by the dentists of the olden times. I heard Dr. Wedelstadt last December in Columbus, express in his most impressively dramatic manner that he uncovered his head and bowed with reverance to the memory of the old soft foil operator of forty years ago. He said it filled him with chagrin that he was compelled to admit that he himself could not, by any effort, acquire the skill that was displayed by these soft foil men. It is like a lost art.

If then, the number and kinds of buccal and dental diseases were the same, and if extraordinary skill was employed in the manipulative treatment of these diseases and deformities with bridges, fillings, crowns and plates, if, in other words, operative dentistry, orthodontia, prosthesis and surgery were practiced fifty years ago in fine style, what changes in modern practice are due to the advancement of science instead of to the improvement of machinery?

We live in a scientific age. How has this science affected the practice of dentistry? I shall try to answer this question by claiming that the *principles* of practice have been placed upon an entirely different basis from that of the early practice—and this even when operations are identical. This proposition I shall try to defend by the following illustrations:

The early dentist removed carious dentin and shaped a cavity, and filled it with his incomparable Abby's Foil for the sole purpose of combating dental caries and stopping the cavity. He did not know the etiology of caries, though he believed then that a perfectly clean tooth could not decay. The microscope had not exposed the bacteria, nor had Pasteur proved the relation of micro-organisms to fermentations and specific diseases. But what difference did this make in practice, provided the dentist excluded the unknown cause and arrested caries? This is not where the change in principle occurs. It is in this the dentist of to-day does not fill teeth simply to stop decay. He labors for the restoration of contour and form of the tooth, and for its ideal occlusion. He does this because the tooth is a part of an organ and not an organ itself. This organ embraces among its functions not only mastication to which the nutrition of the entire body is related, but respiration in a more round about way and voice in a distinct way. He considers broad principles of function. The flat, permanent stoppings of the part have given place to the mesio-distal restoring and the cusp restoring shapes of teeth as units of an important whole. This one principle alone based on increased knowledge of general physiology and pathology, and on the knowledge afforded us by the science of other medical specialists, has so modified the practice that it is not the same thing to-day to fill a tooth that it was fifty years ago. The principle underlying this one operation is new. The reasons for performing the operation have so broadened, that caries is only one of the things considered when we fill a tooth. We now regard the nutrition of the body—the health of the gums, the prophylaxis of the mouth, the preservation of the festoons of the gums, and I am almost tempted to say, the structural development of the jaws of following generations.

I do not believe that as much skill is required to-day to fill teeth as was shown by fine operators a half century ago. The improved tools, the rubber cloth and the electric engine have removed many of the serious obstacles that were always in the way of the older operator. The work has been simplified, thereby reducing the necessity for the cultivation of a high degree of skill.

A second point which I should like to emphasize is the change of practice in the treatment of interstitial gingivitis. The exhaustive histological work done on the tissues of the mouth has opened our minds to a far better understanding of histo-pathology. The physiology and pathology of one membrane—the pericemental, is so clear to-day that treatment for cure and prevention of inflammatory affections of this vital attachment structure and vascular nourisher of the teeth, occupies a large share of our time and skill.

The surgery connected with this membrane is no longer a pioneer work. It is the result of the accumulated experience of a score of years. We now spend hours and weeks in treating, where a few seconds with the forceps completed the cure in the older days. This has required a rearrangement of our ideas about time, fees and general practice.

In this, too, we have had to combat the preconceived inherited notions of patients and dentist. Such changes are difficult. The man who has always had free passes to the theatre and on railroads is less inclined to appreciate the necessity and justice of having to pay for these privileges. From the custom of the older dentists to make no charge for simply cleaning the teeth, or to make a nominal fee only he placed his patients on the free list as far as this operation was concerned. To-day, we suspend the free list for the thorough cleaning of the roots and crowns, which means the most delicate and difficult surgery which the dentist is called upon to perform, may be the specialty of a practice. A half or a third of each day's service can be devoted to this one operation with the best of scientific reasons for a backing, and the best results as an end for present and future health of the teeth and of the patient. Certainly professional fees should keep pace with the importance of the operation. The treatment of the periodontal membrane is recognized by patients as legitimate practice. It is spoken of and demanded by them. How often are we told by patients that so and so has Rigg's disease. Knowledge of these things grows little by little, just as the knowledge of appendicitis and the mastoid operation has with the public. We know that increased knowledge of histology has initiated this change in our daily dental practice. When we remove calcarious deposits from the teeth and polish the exposed surface and stimulate by massage,the gingivae, we are doing all this for broader reasons than the dentist of fifty years ago knew about. Therefore we now insist upon monthly or bi-monthly appointments with our patients and are adding to our list of regulars who are called by telephone for this prophylactic attention. This is a decided change from older notions of practice. I have, as you know, claimed that there is room for assistance in this department of dental practice. I should like to have a class of trained women who would polish teeth and massage gums weekly or fortnightly or at least monthly for children, youths and refined adults and bear the same relation to the dental surgeon that the trained nurse and masseur does to the general physician and surgeon. Orthopoedic surgeons employ women assistants who have been regularly trained as gymnastic teachers to direct exercises for the strengthening of weak or atrophied muscles in their little crooked patients. Also women, proficient in the art of massage to apply manual force on deformed patients for the stimulation of contractility in muscle circulation in capillaries, and for the various conditions in which irritations scientifically directed, will arouse vital activity in wasted or wasting tissue.

The occulist must have his private hospital and trained nurses or fall behind the demands of the times. When we study Talbot of our own profession and other writers on the evolutions and degenerations of tissues, their origins and causes, we are impressed with the scientific value of the proposition that the stimulation of an organ, by normal use or function, is necessary for its proper development and continued health. It is made as vividly clear to us also that lack of use surely results in degenerations, either by atroply or by metamorphose of cells, causing final necrosis of some of the more transitory structures. These conditions are not temporary or observed simply in the life of an individual, put as it has required ages to develop the structure in the perfect form, so the lack of use in preceeding generations has increased a hundred fold the tendencies toward imperfect development or degenerating types in ours.

The law of inheritance is brought into prominence. This accepted science of our day has changed the character of orthodontia practice. Instead of extracting sixth-years molars and bicuspids, as the dentist of old did, to make space for the re-arrangement of the incisors for appearance sake only, such men as Angle, Case and others too numerous to mention, are struggling with entire dentures and maxillas to restore harmonious facial and functional arrangement. This is a higher orthopoedic measure than the mere straightening of irregular incisors. In connection with this subject I have long felt that we should begin these orthopoetic efforts with infants before the eruption, in many cases, of the earliest incisors.

The infantile habit of sucking the thumb produces marked protuberance of the anterior segment of the maxillary arch. Adenoids, which sometimes arises from the failure of the third tonsils to follow its natural course and degenerate by atrophy, deflected septæ in the nasal tract and some other mal-formations of the upper respiratory passages, cause open mouth breathing and consequent persistent lateral pressure by the muscles of the cheek on the growing maxilla of the infant. This interrupts or interferes with natural development and produces narrow arches and mal-position of the adult teeth. It being true, then, that continued slight pressures in infancy anteriorly and outwardly as in thumb sucking, and inwardly and laterally as in open mouth breathing do produce mal-formations, certainly a wisely directed system of manipulation by the fingers of the trained dental nurse or of the dentist (if he will take this practice) in the opposite directions, would expand the growing arch and guide the developing structures of the jaws along the lines of ideal form.

We have all seen what the anxious and intelligent mother can accomplish for her child by finger pressure, even in torsion, in a single refractory incisor when she has been taught by the dentist how to apply the force.

The people of this age appreciate their teeth and the value of dental services. Millions of dollars are annually spent in America alone in efforts to keep the teeth. All classes, rich and poor admire perfect teeth, and if the dental profession would promise to produce fine arches and pretty teeth by simple, painless manipulative measuers in infancy, the demand for just such orthopoedic services would increase beyond our most sanguine calculations.

It would become popular with the public for the above reason alone. These services would be also directly in keeping with our highest principle of dental prophylaxis. This view of the orthodontia and the prophylaxis of the future shows the importance of the kind of service which would be rendered by the women sub-specialist under the scientific dentist's careful supervision.

To take up another question of my subject, let me say, there has been within a few years past advances made in physiologic knowledge in relation to the nutrition of the body. The subject of buccal, gastric and intestinal digestion and their relative values in the economy of the organism has been carefully studied by laboratory experimental methods, and mastication, preliminary to deglutition has come to the front as an important factor, for two reasons. First, that thorough mastication of our cooked starchy foods, with the accompanying more perfect insalivation assists in the digestion of these substances, because even after the bolus enters the acid gastric juice of the stomach, the ptyalin fermentation continues for some time. Second, that this thorough mechanical grinding of all particles of food by prolonged mastication, reduces the quantity and changes the condition of coarse and useless debris, which, finding its way into the lower bowels, makes of this simply a dumping ground for useless food stuffs. This refuse becomes as offensive as does the garbage dump on a city lot. It affords, too, a fine feeding and breeding ground for incalculable myriads of rapidly multiplying fungi. The eminations and poisonous products resulting from the activity of these bacteria are a constant menace to the health and even life of the human being. The toxic matter produces inflammations of the lining membranes of the intestines escaping and poisoning of the blood. Modern science has clearly shown the cause of auto-intoxications and septic infections many of which result in serious acute, distressing and dangerous chronic diseases. We can almost agree with Melchinkoff in his prophecy that the surgery of the future will include the operation of removing, for sanitary reasons, a large portion of this dumping tube.

Sir Michael Foster and other distinguished physiologists have lately been deeply interested in experiments proposed by Horace Fletcher, which tend to prove that prolonged and thorough mastication of food stuffs will so dimish the quantity of debris, that the lower bowel will be changed from a dumping ground to a simple passage for normal waste products. Dentistry is particularly interested in the experiments now being made in physiological laboratories along this line for improved mastication must imply a perfect apparatus, and our modern orthodontia and operative dentistry becomes of vastly increased value to the people. Not for asthetic reasons alone, but as prophylactic against imperfect general nutrition and positive disease from auto-infections. This changes again our principles of practice of accentuates the importance of our professional services. It broadens our field and draws us directly into the enclosure of medical practice, whether we will or not.

There is one other point which I think will help us in our contrast of the old and the new principles of practice of dentistry on account of science we are becoming more and more concerned with the relations between buccal secretions and the general health. More concernes with the relation of conditions of the mouth with membranes and organs remote from the oral cavity. There is a little book of thirty pages published by Cassell & Company in 1901, by William Hunter, M. D., F. R. C. P., of the London Fever Hospital and Charing Cross Hospital, on the subject of "Oral Sepsis" as a cause of "Septic Gastritis." "Toxic Neurites" and other septic conditions. This book treats in a forcible way of the dangers of septic conditions of the mouth. This little book I had the the pleasure of securing for the Lancet & Clinic, of Cincinnati, and I urged at that time its careful perusal by every medical practitioner.

Dr. Hunter lays great stress upon the fact that medical men overlooked the importance of oral sepsis, while treating with medicines, diet and change of climate, certain persistent catarrhal troubles of the stomach and intestines of the larynx, pharynx and tonsils and bronchial tubes, of skin trouble, nervous exhaustions, etc. He criticises the surgeon who is punctilious to a degree—and most rightly so—in seeing that, so far as local scrubbing and disinfection can effect the result, no single septic organism shall remain in the portion of skin upon which he operates, to contaminate the wound; who regards and rightly so, even one drop of pus in connection with a wound as an evidence of sepsis and of partial failure on his part to attain the perfection of results. The surgeon's whole life, it may be said, is passed in excluding and combating septic infection even in its slightest degree; he also, without hesitation, will perform the most complicated and severe operations, *e. g.* on the stomach or intestinal canal without the slightest regard to the presence of septic teeth, septic roots, septic condition of the gums and buccal membranes of his patient. I have quoted this sentence about oversight of the surgeon from Dr. Hunter's book and if you will permit, I will quote his criticism of the dentist.

"The dentist who does so much for his patient in these days of conservative dentistry and high professional dental skill, who sees so much of the unhealthy oral conditions connected with dental caries and necrosis, who, on the strength of his experience, can reproach the physician for his neglect of such conditions; he also will skilfully gold-cap a tooth on put on a gold bridge, or supply a patient with tooth plates—the gold cap to cover a diseased and blackened tooth, the bridge to form a compact and inaccessible pocket for the growth of pus organisms between itself and the gum, the tooth plate to be worn for years without any cleansing other than scrubbing and as often as not covering foul septic necrotic stumps. Plates so ill-fitting that rather than to be troubled with their removal, the patient allows them to grow into the gums."

In regard to this opinion of the doctor about the dentist I think I am justified in claiming that this reckless disregard of the septic conditions of the mouth is not recognized as modern dentistry. It receives the condemnation of the majority of dentists of to-day, because of the advance in knowledge in these matters of sepsis and sterilization.

A few days ago a jury in one of the courts of Cincninati adjudged damage to the sum of $3,500 against a dentist in favor of a patient who had blood poisoning and necrosis following the extraction of a tooth. The grounds of the award were that the dentist had used a dirty pair of forceps which he took out the pocket of a dirty operating coat. This would, in the minds of all of us, be a sufficient reason for the decision of the court on general principles of decency, but we know that if the forceps had been thoroughly sterilized and taken from a sterilized pocket of a sterilized office coat and used by clean hands with steril finger nails, or by a dentist with a shaven face all of which we subscribe to septic poisoning might have followed if perfect removal of septic matter from the tissue to be operated upon, had not been attained. If the dentist had neglected this one point in cleanliness and sterilization, viz, the aseptic treatment of the patient's mouth before operating, he would be guilty of criminal negligence. This is another change in the daily practice of the dentistry of to-day, which modern science has made necessary. This was beyond the comprehension of the most dainty of the older practitioners. We find, then, that even in the most frequent operation of fifty years ago, a change in practice has been found necessary and that extraction has ceased to be simply a skillful yanking and jerking of a tooth and has passed securely into the realm of aseptic minor surgery. The principles underlying this operation has changed. This demands more time and care and scientific study on the part of the modern dentist aside from the actual finger craft.

